# Decoupled timescales of organic carbon and phosphorus recycling in the global ocean

**DOI:** 10.1073/pnas.2514991123

**Published:** 2026-02-17

**Authors:** Megan R. Sullivan, François W. Primeau, Hojong Seo, Judith Camps-Castellà, Keisuke Inomura, Adam C. Martiny

**Affiliations:** ^a^Department of Earth System Science, University of California, Irvine, CA 92697; ^b^Graduate School of Oceanography, University of Rhode Island, Narragansett, RI 02882; ^c^School of Earth and Environmental Sciences and Research Institute of Oceanography, Seoul National University, Seoul 08826, Republic of Korea; ^d^Department of Evolutionary Biology, Ecology and Environmental Sciences, University of Barcelona, Barcelona 08028, Spain; ^e^Department of Ecology and Evolutionary Biology, University of California, Irvine, CA 92697

**Keywords:** carbon sequestration, nutrient cycling, stoichiometry, biological carbon pump, marine carbon dioxide removal (mCDR)

## Abstract

The ocean’s biological carbon pump exports atmospheric CO_2_ to the deep ocean, where it can remain sequestered for decades to centuries, and attempts to artificially enhance this natural carbon sink by fertilizing portions of the open ocean could help mitigate the impacts of excessive anthropogenic CO_2_ emissions. However, differences in the cycling rates of carbon and other nutrients may impact the long-term response to ocean fertilization. In this study, we use a steady-state global biogeochemical inverse model, optimized to match hydrographic observations, to examine how differential production, remineralization, and circulation-driven re-exposure timescales of organic carbon and phosphorus affect long-term carbon sequestration. We partition global organic matter production based on the time required for regenerated carbon and phosphorus to return to the ocean surface. We find that less than 15% of total organic carbon and 31% of total organic phosphorus production remains sequestered in the ocean interior for ≥1 y, with only 3.3% (1.8 Pg C y^−1^) and 8.3% (0.046 Pg P y^−1^), respectively, remaining for a century or longer. The C:P ratio of the sequestration flux declines with increasing residence time, from 255:1 for total production to 98:1 for material sequestered for 100+ years, indicating that carbon is recycled to the surface more rapidly than phosphorus. This decoupling between carbon and phosphorus sequestration timescales could result in a “productivity hangover,” where the slow recovery of surface phosphate leads to a long-term suppression of global productivity, reducing the net removal of atmospheric CO_2_.

## Significance

The ocean plays a critical role in regulating atmospheric carbon dioxide through the biological carbon pump, which transports organic carbon from surface waters to the deep ocean. However, the efficiency of this process is influenced by the cycling of other essential nutrients, such as phosphorus. This study demonstrates that carbon and phosphorus have distinct residence times in the ocean, challenging assumptions about how much carbon remains sequestered over climate-relevant timescales. Our results suggest that assessments of proposed marine carbon dioxide removal strategies, such as ocean iron fertilization, may be inaccurate if they fail to account for nutrient cycling.

Atmospheric carbon dioxide (CO_2_) concentrations continue to rise due to human-driven disruptions to the global carbon cycle, with far-reaching consequences for climate, ecosystems, and society (1). While rapid decarbonization remains essential to mitigating these impacts, growing attention is turning to carbon dioxide removal (CDR) as a way to offset residual emissions or to limit long-term warming from past emissions. Marine CDR (mCDR) refers to deliberate interventions that enhance the ocean’s natural CO_2_ uptake, encompassing a range of biological and chemical methods (2, 3). Among the most studied methods is ocean nutrient fertilization, particularly with iron (4–8). This approach aims to stimulate phytoplankton growth in nutrient-limited regions, and thereby enhance the ocean’s biological carbon pump—that is, the process by which photosynthetic organisms draw down CO_2_ in the surface ocean, then transport a portion of this carbon to depth where it can remain sequestered for decades to centuries (9). If successful, such biological mCDR strategies could create a substantial, climate-relevant sink for atmospheric CO_2_.

Despite its potential, biological marine carbon dioxide removal methods face significant uncertainties that limit our ability to predict its long-term effectiveness. A central challenge is determining how long the additional organic carbon remains sequestered in the ocean interior, isolated from atmospheric exchange. Only a tiny fraction of organic carbon production is ultimately buried in seafloor sediments (10); the vast majority is remineralized into CO_2_ as it sinks through the water column. This regenerated CO_2_ is eventually returned to the surface by ocean circulation, where it can re-equilibrate with the atmosphere. For ocean-based CDR strategies that rely on enhancing the biological carbon pump to be viable climate mitigation tools, the sequestered carbon must remain out of contact with the atmosphere for climate-relevant timescales—typically decades to centuries (2, 3). Quantifying this sequestration timescale is therefore critical to evaluating the durability, or permanence, of mCDR. However, tracking the fate of exported carbon over time is technically challenging, and few studies have estimated the amount of biologically exported carbon that remains sequestered on century timescales (11). The duration of sequestration depends on the depth and location of remineralization: Shallow remineralization tends to return carbon to the atmosphere more quickly, while deeper remineralization leads to longer sequestration (12). Many studies use the export flux through a fixed depth or isopycnal surface, commonly 1,000 m, as a proxy for century-scale sequestration (13, 14). However, recent work shows that remineralization shallower than 1,000 m can still contribute substantially to long-term carbon storage, with approximately half of biogenic carbon stored for 100+ years originating from shallower depths (15).

Interactions between nutrient and carbon cycles also contribute to uncertainty in evaluating the long-term carbon storage potential of nutrient fertilization as a means of mCDR. By stimulating primary production, fertilization alters the biological uptake and export of other nutrients. The resulting redistribution of nutrients can affect future productivity both locally and in downstream regions (16). Thus, assessing the long-term effectiveness of nutrient-based mCDR strategies requires tracking the fate of both carbon and nutrients. However, this task is complicated by the nonlinear relationships between carbon and nutrient cycles. Phytoplankton exhibit substantial regional variability in elemental composition, particularly in carbon-to-phosphorus (C:P) ratios (17, 18). Differential sequestration rates can arise when such regional variations in C:P stoichiometry coincide with differences in circulation-driven re-exposure timescales, because areas with carbon-enriched organic matter export and slower physical ventilation retain carbon longer. Additionally, the remineralization rates of carbon and nutrients need not be identical, nor uniform in space. For instance, microbial communities may preferentially remineralize phosphorus-rich compounds (19–21) or vice versa (22), resulting in different remineralization depths, and therefore different sequestration timescales, for carbon and phosphorus.

To test how differential carbon and phosphorus cycling affects long-term carbon sequestration, we used a steady-state global biogeochemical inverse model with variable export pathways for organic carbon and phosphorus. The model was designed to simulate the export and remineralization of dissolved and particulate organic matter, partitioning these export fluxes by their residence time in the ocean interior, i.e. the amount of time the exported material remains stored beneath the sunlit surface layer of the ocean, before next coming into contact with the atmosphere. Our modeling approach uniquely allows us to assess the residence time of regenerated carbon and nutrient fluxes, moving beyond a depth-based carbon export framework to directly evaluate carbon and nutrient sequestration in the ocean interior. We quantify how differences in organic carbon and nutrient cycling rates influence the long-term response to changes in surface conditions, such as those induced by climate change or artificial nutrient enrichment. Our findings show that organic carbon and phosphorus sequestration fluxes have distinct recirculation timescales. Furthermore, the carbon to phosphorus stoichiometry varies depending on the timescale of sequestration in the ocean interior, indicating that measuring euphotic zone stoichiometry is insufficient to fully characterize carbon and nutrient sequestration on climate-relevant timescales.

## Results

### Partitioning Organic Carbon Production by Residence Time.

We partitioned global organic matter production according to its residence time in the ocean interior, demonstrating that a substantial fraction of organic matter production is rapidly recycled back to the surface. Let τ denote the first-passage time of regenerated dissolved inorganic carbon (DIC_bio_) and phosphorus (DIP_bio_)—that is, the time until their next surface contact—following Primeau (23). We then define biogenic sequestration fluxes, $\Phi_{\tau \geq t}^{\mathrm{TOC}}$ and $\Phi_{\tau \geq t}^{\mathrm{TOP}}$, as the global production of organic carbon and phosphorus whose regenerated inorganic forms remain in the interior for at least a threshold time t. These fluxes are integrated over the entire water column and are independent of any fixed export depth. We choose a time-based horizon for ease of interpretation in marine management, as it directly relates to the longevity of sequestered carbon in the ocean.

**Fig. 1.**
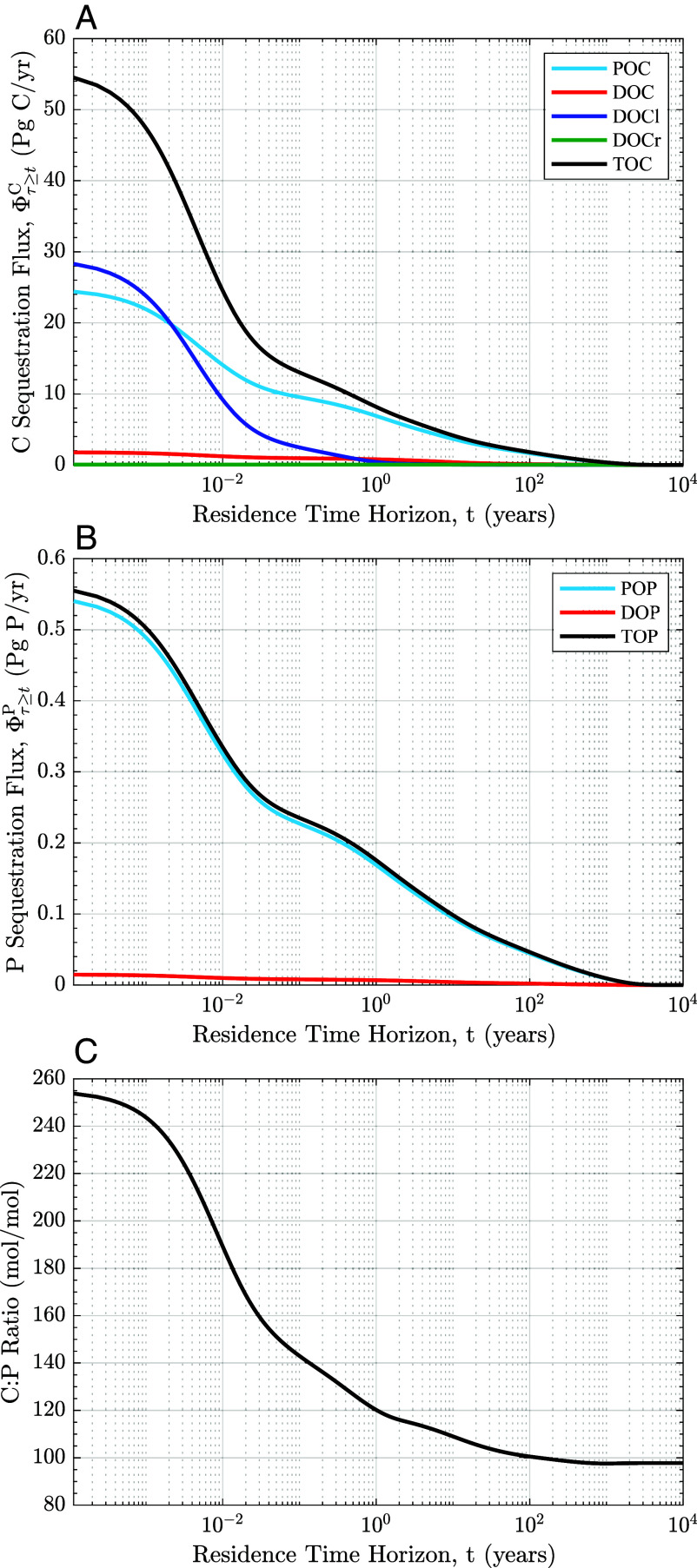
Global sequestration fluxes of carbon ($\Phi_{\tau \geq t}^{C}$) and phosphorus ($\Phi_{\tau \geq t}^{P}$). Sequestration fluxes are defined as the global production of regenerated organic carbon (*A*) and phosphorus (*B*), that remains in the ocean interior for at least t years before next coming into contact with the atmosphere. Panel (*C*) shows the ratio of the total carbon sequestration flux to the total phosphorus sequestration flux ($\Phi_{\tau \geq t}^{\mathrm{TOC}}/\Phi_{\tau \geq t}^{\mathrm{TOP}}$) as a function of the residence time horizon, t. Note that the residence time horizon, on the x-axis, is on a log scale. In (*A* and *B*), total organic carbon (TOC) and total organic phosphorus (TOP) sequestration fluxes (black) are the sum of remineralized particulate organic matter (light blue), semilabile dissolved organic matter (red), labile organic matter (dark blue), and recalcitrant dissolved organic matter (green) production. For a residence time threshold of t=0, the TOC sequestration flux is equivalent to global net primary production (NPP), which is prescribed to match satellite-based climatological estimates from the CbPM algorithm (24).

We find that most biogenic carbon has a short residence time in the ocean interior. Fig. 1*A* shows the sequestration flux as a function of the residence time horizon, t. At t=0, the sequestration flux of total organic carbon (TOC), $\Phi_{\tau \geq 0}^{\mathrm{TOC}}$, is 55 Pg C y^−1^. This flux represents organic carbon remineralization integrated across all residence times and is equivalent to net primary production (NPP). The biogenic carbon sequestration flux with a residence time ≥ 1 y is 8.1 Pg C y^−1^, indicating that less than 15% of TOC produced in the euphotic zone remains sequestered in the ocean interior for at least 1 y. The century scale sequestration flux, $\Phi_{\tau \geq 100\;y}^{\mathrm{TOC}}$ is 1.8 Pg C y^−1^, which is approximately 3.3% of total organic carbon production. These results indicate that much of the organic carbon production is rapidly remineralized and returned to the surface, without contributing to long-term storage.

This rapid loss is driven largely by the production of labile organic carbon. The model is designed to match satellite-based estimates of NPP by adjusting production of a rapidly cycling labile dissolved organic carbon (DOC_*l*_) pool. In the optimized model, DOC_*l*_ constitutes 52% of TOC production globally. However, labile carbon is rapidly remineralized near the surface, with a respiration timescale of 12 h. After one year, only 5% of the remaining sequestered DIC_bio_ originated as DOC_*l*_ (Fig. 1*A*). Thus, while labile carbon contributes significantly to surface NPP, it does not substantially impact long-term carbon sequestration. In contrast, particulate organic carbon (POC) has a longer residence time and dominates sequestration fluxes on annual to centennial timescales. These contrasting sequestration flux curves highlight how different organic matter pools contribute to sequestration over different time horizons.

The sequestration flux of organic phosphorus is driven primarily by the production of particulate organic phosphorus (POP). Unlike the carbon cycle model, which includes an explicit labile DOC pool, all advective–diffusive organic phosphorus fluxes are represented by a single dissolved organic phosphorus (DOP) tracer. This simplified formulation reflects the limited observational constraints on organic phosphorus cycling; the sensitivity to this assumption is addressed later in the text. At t=0, the phosphorus sequestration flux, $\Phi_{\tau \geq 0}^{\mathrm{TOP}}$, is 0.56 Pg P y^−1^. About 31% (0.17 Pg P y^−1^) of total DIP_bio_ production remains sequestered in the ocean interior for 1 y or longer. For a residence time horizon of 100 y, the sequestration flux, $\Phi_{\tau \geq 100\;y}^{\mathrm{TOP}}$, drops to 0.046 Pg P y^−1^, or about 8.3% of the total production (Fig. 1*B*). This residence-time partitioning reveals a biological pump dominated by short-lived fluxes, with only a small fraction of production remaining sequestered on climate-relevant timescales. Notably, a larger share of biogenic phosphorus than carbon remains sequestered after 100 y, indicating that phosphorus has a longer average sequestration timescale than carbon in the model.

### C:P Stoichiometry Across Residence Time.

The biogenic sequestration stoichiometry varies across residence time horizons (Fig. 1*C*). In our optimized model, the global average carbon-to-phosphorus (C:P) ratio of total organic matter production is significantly higher than the traditionally assumed “Redfield” proportions of 106 mol C : 1 mol P (25). The ratio of $\Phi_{\tau \geq 0}^{\mathrm{TOC}}$ to $\Phi_{\tau \geq 0}^{\mathrm{TOP}}$ exceeds 250:1 (Fig. 1*C*, y-axis intercept), reflecting carbon-enriched organic matter in the surface ocean. However, the C:P ratio decreases with increasing sequestration timescale. The C:P ratio of the biogenic sequestration flux with residence times longer than about 1 mo drops to 145:1, and further decreases to about 120:1 for the sequestration flux with residence times of at least one year. On century timescales and longer, the ratio declines to approximately 98:1. The differing stoichiometry of the biogenic sequestration flux as a function of residence time indicates a decoupling of the organic phosphorus and carbon cycles, in which organic carbon and nutrients have different recirculation timescales.

The relationship between biogenic carbon and phosphorus sequestration shows contrasting behavior between short- and long-term residence time horizons. When considering the sequestration fluxes across short residence time horizons (<1 y), the shallow remineralization and rapid resurfacing of regenerated labile DOC causes a steep decline in the TOC sequestration flux. Meanwhile, the phosphorus sequestration flux, which is driven primarily by the inventory of regenerated POP, declines more gradually. Consequently, over sequestration horizons ranging from seconds to months, the C:P ratio declines sharply as residence time increases. For longer-term sequestration, the change in stoichiometry across residence time horizons is dampened. Although labile organic carbon makes up over 50% of surface TOC production, by a residence time horizon of 1 y, it supplies only 5% of the carbon sequestration flux, while particulate organic carbon (POC) constitutes 85% of the sequestration flux (*SI Appendix*, Fig. S2). At century-timescales, more than 90% of the sequestered DIC_bio_ and DIP_bio_ originates from particulate organic matter. Across long residence time horizons, where the sequestration flux is insensitive to labile organic matter production, the more gradual decline in C:P stoichiometry with residence time can be attributed largely to differences between POC and POP production and remineralization.

**Fig. 2.**
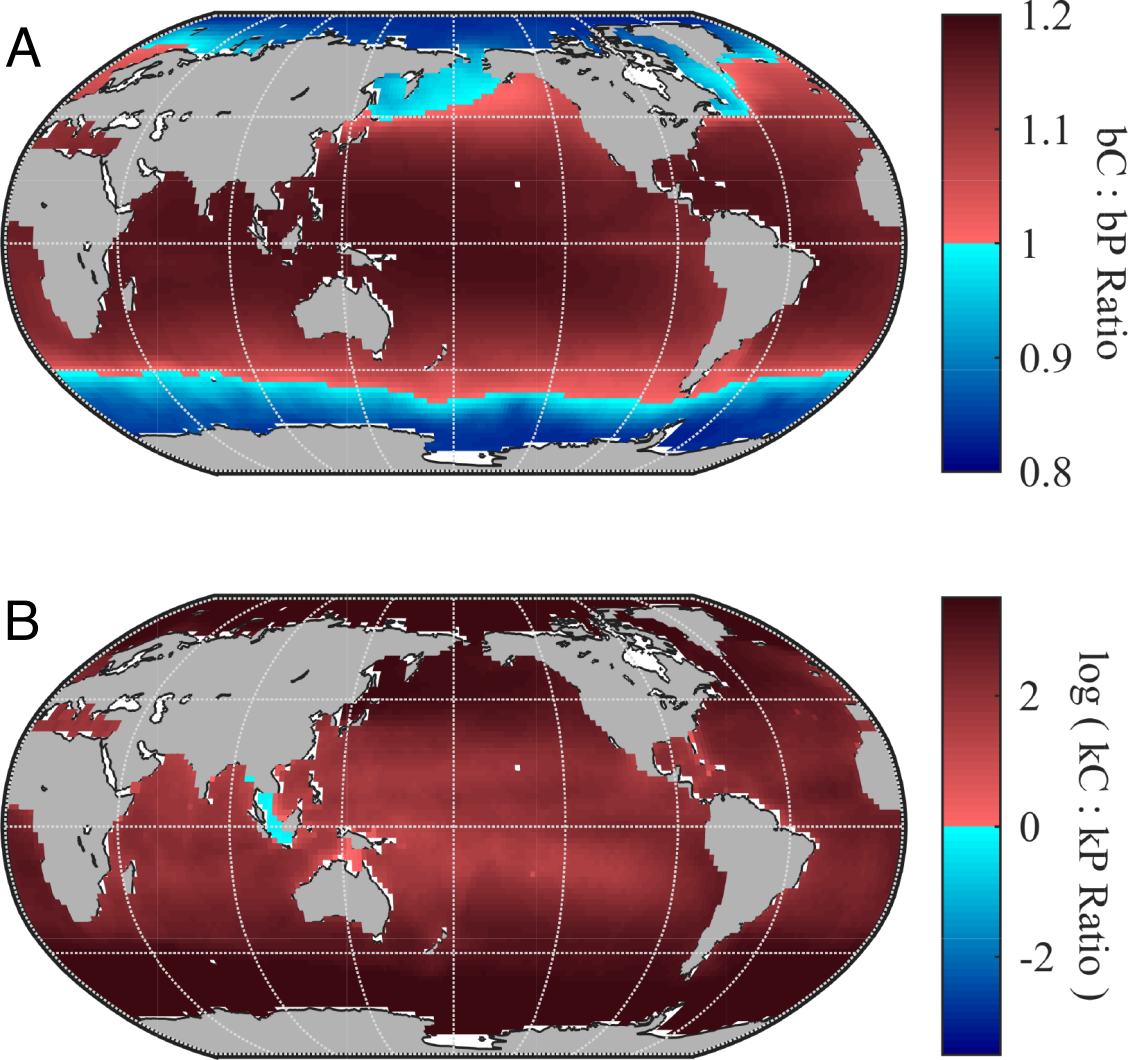
Comparison of remineralization rates of organic carbon and phosphorus. (*A*) The ratio of optimal exponent “b”-values for the vertical flux attenuation of particulate organic carbon (POC) and phosphorus (POP). Particle flux attenuation profiles are parameterized following Martin et al. (26). In (*A*) values greater than 1 (red) indicate that POC attenuates faster with depth (i.e. shallower remineralization) than POP; blue indicates that POP attenuates faster with depth than POC. (*B*) The ratio of the water-column average remineralization rate constants for semilabile DOC ($k_{C}$) versus semilabile DOP ($k_{P}$). The ratio is log transformed to emphasize regions where the ratio is near 1. $\log(k_{C}:k_{P})>0$ indicates preferential remineralization of DOC relative to DOP. Vertical averages are weighted by DOM remineralization.

In our inverse model, organic carbon remineralizes faster than organic phosphorus across much of the ocean. Fig. 2*A* illustrates the relative particle flux attenuation coefficients ($b_{C},b_{P}$) and remineralization rate constants ($k_{C}$, $k_{P}$) for organic carbon and phosphorus. These patterns emerge from our biogeochemical inverse model optimization, which finds the set of biogeochemical parameter values that minimize the misfit between model and observed global inorganic tracer fields (*Materials and Methods*). The optimized parameters control the depth attenuation of POC and POP fluxes, as well as the timescales for semilabile DOP and DOC remineralization. Both particulate and dissolved organic matter remineralization are temperature-dependent, with each tracer exhibiting a unique temperature sensitivity. In the optimized model, POC attenuates faster with depth than POP over most of the ocean, with the exception of subpolar and polar regions (Fig. 2*A*). Additionally, the timescale for DOP remineralization is much longer than for DOC, with flux-weighted global averages of 101 y (range: 4 to 1,070 y) for DOP and 10 y (range: 5 to 19 y) for semilabile DOC. The rate of DOP remineralization only exceeds that of DOC remineralization at shallow depths near the equator, while in the rest of the ocean, the model finds preferential remineralization of DOC relative to DOP (Fig. 2*B*). These results both indicate faster cycling of organic carbon than phosphorus and contribute to the observed decline in C:P with longer-term sequestration.

Spatial variability in the stoichiometry of surface production also contributes to the change in C:P across residence time horizons. To separate the effects of remineralization from surface production patterns, we conducted a sensitivity test in which carbon and phosphorus were assigned identical remineralization parameters: $b_{C}=b_{P}=1.28$ and $k_{C}=k_{P}=0.057$ y^−1^, equal to the flux-weighted global means from the optimized model. In this configuration, the C:P ratio still declines with residence time, but the trend is less pronounced. The C:P ratio of total regenerated production ($\Phi_{\tau \geq 0}^{\mathrm{TOC}}$ : $\Phi_{\tau \geq 0}^{\mathrm{TOP}}$) drops from the optimized model result of 255:1 to 225:1, and the century-scale sequestration flux C:P ratio rises from 98:1 to 126:1 (*SI Appendix*, Fig. S1). This dampening confirms that differential remineralization is a significant driver of the steep decline in C:P with increasing sequestration time. However, the persistence of the declining trend, even with uniform remineralization, indicates that spatial patterns in surface production stoichiometry—particularly, the colocation of regions with low production C:P and regions where the physical circulation produces long residence times for the regenerated material—also play a significant role.

### Sensitivity to Fast-Cycling Labile Organic Phosphorus.

**Fig. 3.**
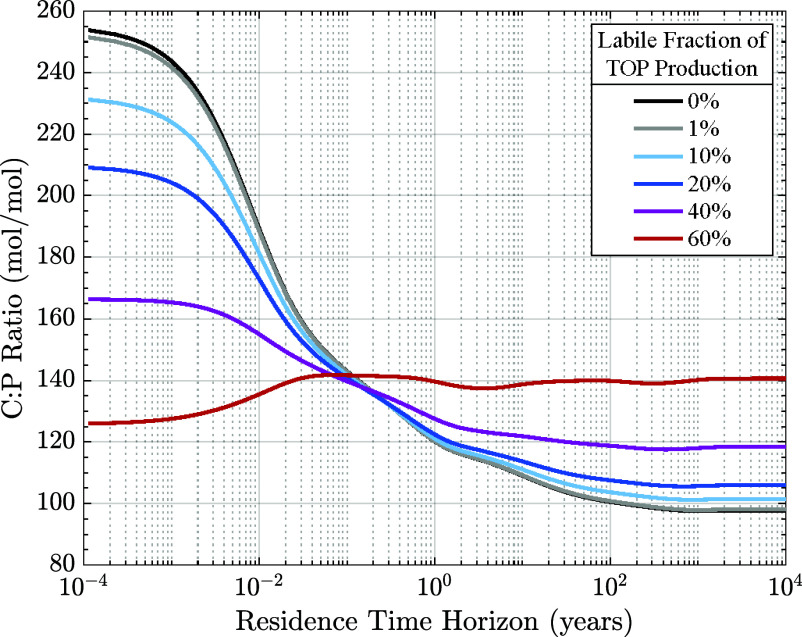
Model sensitivity to fast-cycling labile organic phosphorus. The global mean ratio of residence-time-partitioned total organic carbon to phosphorus production for a range of simulations, testing the sensitivity to fast-cycling labile organic phosphorus production in the model. The phosphorus cycle was recomputed with varying fractions of total organic phosphorus production routed to an added labile DOP pool (1%, 10%, 20%, 40%, and 60%). The carbon cycle remains fixed across all simulations. While the optimized model with no labile DOP provides the best fit to hydrographic tracer observations, all models reproduce observed phosphate concentrations well. The R^2^ fit to GLODAPv2 phosphate concentrations (27) is about 0.92 for the optimized model and the sensitivity tests with 1%, 10%, and 20% allocations to labile DOP. For the 40% and 60% labile DOP models, the R^2^ fit drops to 0.90 and 0.84, respectively.

Varying the fraction of labile DOP (DOP_*l*_) highlights how changing the balance of fast-cycling phosphorus versus carbon can alter the C:P ratio for short- and long-residence fluxes. The elevated carbon to phosphorus (C:P) ratios of total surface production are largely driven by the production of labile organic carbon. Labile production has minimal impact on deep-ocean tracer fields. Consequently, inversions based on interior hydrographic observations do not effectively constrain these fluxes. To test the sensitivity of the residence-time-partitioned sequestration flux stoichiometry to the model’s handling of labile organic matter, we recomputed the equilibrium phosphorus cycle after routing different shares of the total organic phosphorus production, namely 1%, 10%, 20%, 40%, and 60%, into an added labile pool. DOP_*l*_ is assigned the same 12-h lifetime as labile DOC. Introducing DOP_*l*_ to the phosphorus cycle, while maintaining the previously optimized parameter values, reduces the particulate fraction of organic phosphorus production, since the model’s POP production is determined from the residual after subtracting fixed fractions allocated to semilabile and labile DOP production. In each of the new phosphorus cycle equilibrium states, the increased production of DOP_*l*_ leads to an increase in the total surface production of organic phosphorus. However, this also results in a decrease in the 1 y phosphorus sequestration flux, $\Phi_{\tau \geq 1\;y}^{\mathrm{TOP}}$. This occurs due to the redistribution of total production from the longer-lived particulate pool to the fast-cycling labile pool. We then calculated the residence-time-partitioned sequestration of organic phosphorus in these new steady-state solutions and used it to determine the C:P ratio of regenerated production as a function of residence-time. For these calculations we held the model’s carbon cycle fixed to its optimized solution, in which 52% of surface TOC production was allocated to the labile DOC pool. The resulting C:P ratios are plotted against residence time in Fig. 3.

In simulations with up to 40% of TOP production routed to DOP_*l*_, the general trend of decreasing C:P ratios with increasing residence time is maintained, but the magnitude of this change is dampened (Fig. 3). This effect becomes more pronounced with higher allocations to DOP_*l*_. The trend is only reversed when more than half of organic phosphorus production is routed to the labile pool. With 20% of organic phosphorus routed to DOP_*l*_, the molar C:P ratios range from 210:1 for rapidly recycled material to 106:1 for material sequestered for centuries or longer. Both ends of the range move closer to the mean: Short-term C:P ratios decrease, while long-term ratios increase compared to the base model. With 40% of phosphorus production routed to labile DOP, the range narrows further, with C:P starting at 166:1 for short residence times and decreasing to 118:1 for the longest residence times. In the extreme case where 60% of organic phosphorus production is routed to DOP_*l*_, the C:P pattern across residence times changes significantly. For submonthly timescales, C:P increases with residence time, starting lower than in the optimized model due to the rapid recycling of the abundant DOP_*l*_. Globally, the fraction of labile phosphorus production exceeds that of labile carbon, driving the short-residence-time C:P ratio below the integrated average. For century-scale and longer sequestration, the C:P ratio rises to 141:1, higher than in the base model. In this model, long-term sequestration is more carbon-enriched. With less POP reaching the deep ocean compared to the unaltered POC export, the overall C:P ratio increases at long residence time horizons.

While this 60% DOPl scenario represents an upper bound in our experiments, it is not inherently unrealistic within the context of our coarse-resolution inverse model. In this framework, “labile” refers to organic matter that is produced and remineralized rapidly enough that it does not influence tracer concentrations beyond its resident grid box. Rapid biological cycles cannot be constrained because their transformation timescales are much shorter than physical transport between coarse grid cells. Our optimized solution routes approximately half of global organic carbon production into the labile DOC pool. If fast-cycling organic matter has stoichiometry similar to bulk organic matter, a comparable fraction of total organic phosphorus production could plausibly be routed into an equivalently fast-cycling DOP pool.

Our results offer an example of the differential cycling of carbon and nutrients, demonstrating that the stoichiometry is not guaranteed to remain constant across residence time horizons. However, because of labile biological fluxes that only affect short-term sequestration, there is significant uncertainty in the magnitude and even direction of change of C:P ratios as a function of residence time. While the model’s ability to reproduce deep ocean inorganic nutrient fields remains intact, assumptions about poorly constrained labile organic matter lead to different C:P relationships for short-term sequestration (Fig. 3). The range of outcomes from this sensitivity test indicates that our assumptions about the rapid cycling of organic matter strongly influence the relationship between organic carbon and phosphorus fluxes as a function of residence time. By changing the proportion of organic phosphorus production that cycles rapidly, we can dramatically change the residence time partitioned ratio of organic carbon and phosphorus production. Therefore, the model results shown here should be viewed as examples, not definitive representations of the real ocean, particularly for sequestration fluxes with residence times less than a year. Nevertheless, the finding that biological sequestration stoichiometry varies across storage time horizons is noteworthy, as it has important implications for the effectiveness of nutrient fertilization as a marine carbon dioxide removal strategy.

## Discussion

### Implications for Artificially Enhancing Surface Production.

Our model finds that, at steady state, the carbon and nutrient recycling pathways are not identical. This has important implications for understanding the net impact of nutrient fertilization on the global ocean carbon sink on long timescales.

The differing cycling rates of carbon and nutrients can lead to significant regional imbalances in ocean productivity following micronutrient addition. When micronutrients like iron are added to stimulate surface production, the induced blooms rapidly consume available nutrients that might normally be transported downstream, reducing the nutrient supply to dependent ecosystems. This could induce or exacerbate nutrient limitation downstream, inhibiting organic carbon production and export there. This possibility, termed “nutrient robbing” (28), has long been recognized as a major challenge to verifying the efficacy of nutrient fertilization, since nonlocal responses may offset any increases in carbon export measured locally at the fertilization site (16). Spatial differences in the relative biological uptake rates of phosphorus and carbon can amplify the impact of nonlocal responses. In a simplified box model, Moreno et al. (29) demonstrated how enhancing organic matter production in regions with low C:P uptake ratios can actually lead to a net decrease in globally integrated carbon export. This happens because the enhanced nutrient drawdown in these regions reduces production and export in areas with higher C:P ratios. Moreno et al.’s analysis focused on the spatial variability, showing how local versus downstream C:P ratios control the net change in ocean carbon uptake caused by fertilization (29). However the long-term response will also depend on how these C:P ratios evolve with time due to the differing remineralization pathways between organic carbon and organic phosphorus.

While our analysis focuses on the coupled cycling of carbon and phosphorus, nitrogen also plays a critical role in regulating ocean productivity and export. Nitrogen typically tracks carbon more tightly than phosphorus during biological uptake, and evidence suggests organic nitrogen remineralizes on shorter timescales than organic carbon (e.g., refs. 19 and 30). Incorporating an explicit nitrogen cycle would likely yield intermediate sequestration timescales between those of carbon and phosphorus, and remains an important direction for future extensions of this modeling framework.

### Productivity “Hangover” After Iron Fertilization.

After a pulse of micronutrient addition, enhanced surface production will initially draw down excess nutrients (e.g., phosphate). Any excess nutrients exported to the deep ocean can no longer support new surface production until they are recirculated back to the upper layers. The expected response to this perturbation depends on the relative cycling rates of carbon and phosphorus. In one scenario, the atmospheric CO_2_ is sequestered for longer, while phosphorus is returned to the surface more quickly. This allows for a faster recovery of surface productivity, making this a potentially effective pathway for long-term atmospheric CO_2_ reduction. This case is seen in the 60% DOP_*l*_ sensitivity test (Fig. 3), where the C:P ratio of stored material increases for longer term storage.

**Fig. 4.**
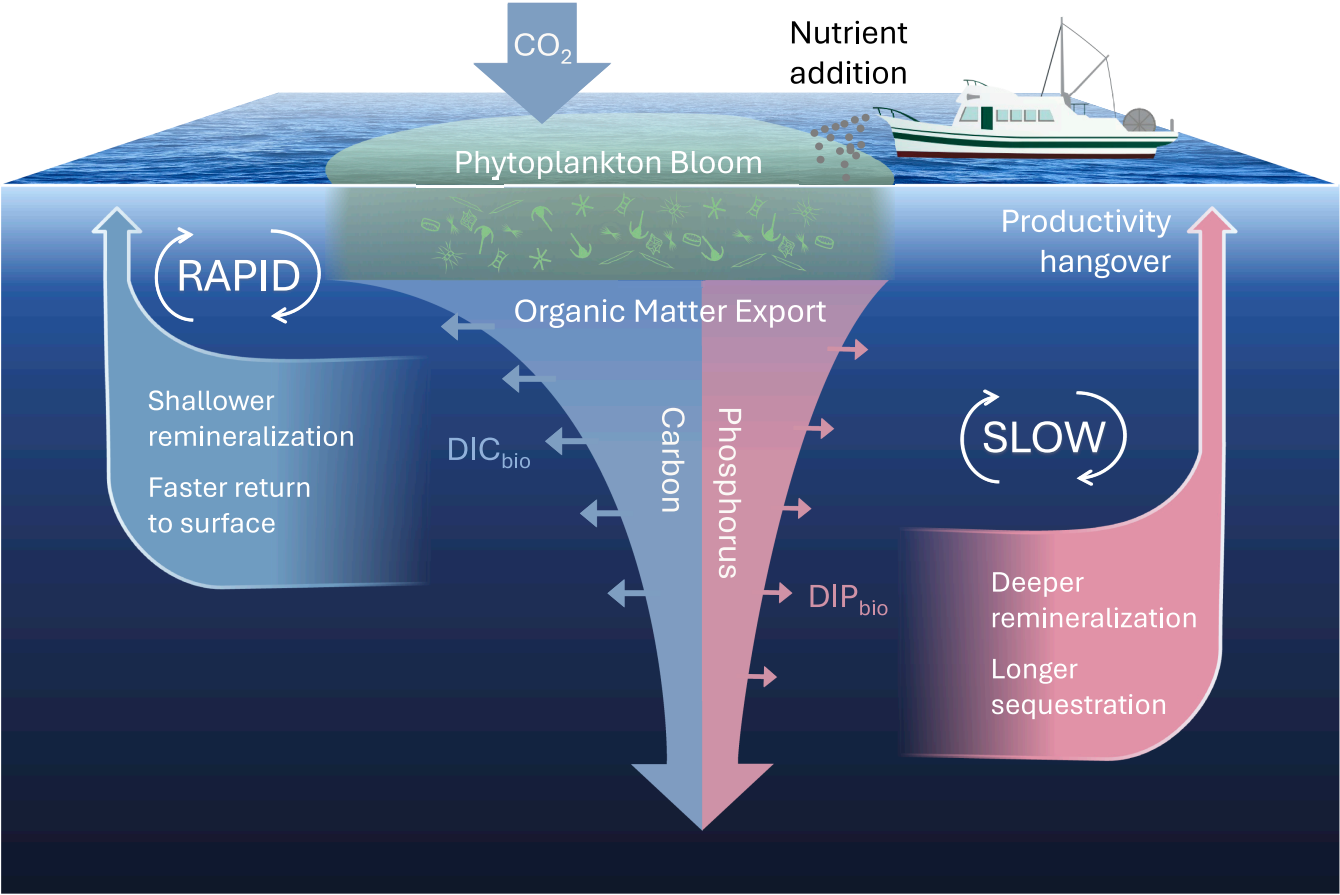
Schematic representation of differential carbon (blue) and phosphorus (pink) cycling following enhanced surface productivity. Our optimized biogeochemical inverse model indicates that organic carbon is recycled more rapidly than organic phosphorus in the global ocean. On average, organic carbon is remineralized at shallower depths, leading to a more rapid return to the surface ocean as dissolved inorganic carbon (DIC_bio_). In contrast, organic phosphorus remineralizes at greater depths, resulting in longer nutrient sequestration and delayed return to the surface as dissolved inorganic phosphorus (DIP_bio_). The slow recovery of surface phosphate following nutrient fertilization may cause a prolonged “productivity hangover,” during which heightened nutrient limitation suppresses total surface production below prefertilization levels.

In contrast, if the C:P ratio decreases for longer-term storage (as is the case for all sensitivity tests with up to 40% of organic phosphorus production allocated to the labile pool), the micronutrient addition would not result in long-term CO_2_ reduction. The conceptual diagram in Fig. 4 illustrates this outcome. Initially, the pulse stimulates production, leading to increased carbon export. However, this exported carbon is quickly recirculated back to the surface, while the excess nutrients, now stored more efficiently in the deep ocean, are unavailable to support future surface production over an extended period of time. This could lead to a prolonged decrease in ocean productivity following the micronutrient addition, lowering the net ocean uptake of atmospheric CO_2_ over time.

In other words, depending on the relative cycling rates of carbon and nutrients, there could be a prolonged productivity “hangover” relative to the initial pulse of carbon export induced by nutrient fertilization, inhibiting the net ocean uptake of atmospheric CO_2_ over time. In one case, the system recovers quickly; in the other, the loss in productivity lingers. The key difference lies in the cycling of phosphorus, highlighting the need to extend marine CDR studies beyond carbon fluxes and consider how the associated nutrient cycles respond to micronutrient addition.

In the model that is best able to reproduce hydrographic observations, the globally integrated regenerated C:P ratio decreases with increasing residence time, suggesting on the global scale, enhancing surface productivity could be inefficient for carbon sequestration on climate-relevant timescales. However, as demonstrated in the DOP_*l*_ sensitivity test, the magnitude and even direction of this effect depend on the relative production of labile organic matter. For example, if nutrient fertilization preferentially stimulates phytoplankton functional groups that produce more labile DOP, the resulting carbon sequestration may be more efficient and less susceptible to a productivity hangover than if the same intervention promotes the production of more persistent POP.

To assess how differential remineralization affects long-term sequestration efficiency, defined as the fraction of total organic carbon (TOC) production that remains sequestered in the ocean interior beyond a given residence time threshold, we conducted a steady-state sensitivity analysis (*SI Appendix*, Fig. S3). In this experiment, we removed the elemental asymmetry by setting phosphorus remineralization parameters equal to those of carbon at every location ($b_{P}=b_{C}$, $k_{P}=k_{C}$). Equalizing these parameters eliminates the slower return of phosphorus to the surface. As a result, phosphorus recycles more rapidly than in the optimized model sustaining higher nutrient availability to support particulate organic matter (POM) formation at steady state. Although total surface carbon production is still prescribed to match satellite-based NPP estimates, the faster phosphorus recycling shifts the organic carbon partitioning toward greater POM production (and reduced labile DOC), thereby increasing carbon export and enhancing long-term sequestration. The impact on sequestration efficiency is substantial. Across 10-, 100-, 200-, and 1,000-y residence time horizons, the optimized model–with relatively slower phosphorus remineralization–exhibits 13.7%, 11.7%, 11.2%, and 9.9% lower TOC sequestration efficiency, respectively, compared to the equal-remineralization case (*SI Appendix*, Fig. S3). To put these differences in practical terms: If ocean iron fertilization were to induce a 1 Pg C increase in global organic carbon production, the equal-remineralization model predicts that 0.0374 Pg C would remain sequestered after 100 y, whereas only 0.0331 Pg C would remain in the optimized model. Thus, sluggish phosphorus remineralization reduces 100-y carbon storage from such a perturbation by approximately 12%.

### Preferential Remineralization.

While much attention has been given to the variability in the C:P uptake ratio of surface production (31–33), it is equally important to understand how organic matter stoichiometry is altered by remineralization processes. The relative remineralization rates of carbon and phosphorus remain debated, with observational evidence supporting both preferential carbon and phosphorus remineralization in the water column. Sediment trap and in situ pump data have shown increasing C:P ratios with depth, suggesting preferential phosphorus remineralization (34, 35). Conversely, particulate organic matter C:P ratios decrease with depth in the Sargasso Sea, indicating faster carbon remineralization (22). While the stoichiometry of suspended POM may incorporate shifts in heterotrophic biomass composition, the observed gradients reflect the net vertical attenuation of organic matter. The former view supports a more efficient biological carbon pump, in which regenerated phosphorus rapidly recycles to maintain surface production, while a greater proportion of organic carbon is transported and stored in the deep ocean. In contrast, our model optimization suggests that organic carbon cycles faster on average than organic phosphorus, consistent with ref. 22. The divergence between model prediction of preferential carbon remineralization and sediment trap-derived evidence of faster phosphorus remineralization may reflect both model limitations and uncertainties in the interpretation of sediment trap data, which are subject to biases such as swimmer contamination, postdeployment remineralization, and undercollection of small or slowly sinking particles (36). Rather than indicating a failure of either model or observation, the mismatch highlights an important uncertainty in our understanding of degradation processes at depth and underscores the need for improved, depth-resolved measurements of organic matter stoichiometry.

In the model, organic matter production in the surface ocean is highly enriched in carbon relative to Redfield proportions. This elevated C:P uptake ratio reflects the combined contributions of particulate, dissolved, and labile organic matter production, each with distinct stoichiometry. As organic matter degrades, its stoichiometry is altered by divergent remineralization pathways for carbon and phosphorus. Our inversion of the hydrographic DIC and DIP observations suggests that in much of the ocean, POC attenuates more rapidly with depth than POP (Fig. 2*A*), and DOC remineralizes faster than DOP (Fig. 2*B*). This preferential remineralization of organic carbon reduces the C:P ratio of global export from the base of the euphotic zone compared to the stoichiometry of production.

The spatial variations in the remineralization rates of organic carbon and phosphorus have significant implications for the design of marine carbon dioxide removal (mCDR) strategies. In regions where POP attenuates faster than POC (i.e., where the ratio of carbon to phosphorus particle flux attenuation coefficients, $b_{C}:b_{P}$, is less than 1), enhanced production of particulate organic matter will be more effective in sequestering carbon relative to phosphorus. Therefore, targeted fertilization efforts in such areas could maximize long-term carbon storage potential. In our model, this condition is met in subpolar to polar regions (Fig. 2*A*), aligning with recent studies highlighting the Northeast Pacific and Southern Ocean as promising areas for ocean iron fertilization as a means of mCDR (37).

Additionally, mCDR strategies that aim to stimulate the production of organic matter with elevated C:P ratios—such as by promoting the growth of carbon-rich macroalgae or phytoplankton—must consider the relative attenuation rates of organic carbon and nutrients, to assess if the high C:P ratios seen in surface production will translate to enhanced long-term carbon sequestration. In regions where $b_{C}:b_{P}>1$, the carbon sequestration potential of a CDR strategy will be overestimated if based on the C:P ratios of POM measured at the surface. Conversely, in regions where $b_{C}:b_{P}<1$, using the surface POM C:P ratios will provide a conservative estimate of long-term carbon storage potential.

Because particulate and dissolved organic matter follow distinct remineralization pathways, effective mCDR strategies must account for both the ratio of carbon to phosphorus particle-flux attenuation coefficients ($b_{C}:b_{P}$) and the ratio of DOC to DOP remineralization rate constants ($k_{C}:k_{P}$). In high-latitude regions, where $b_{C}:b_{P}<1$ and $k_{C}:k_{P}>1$, the C:P ratio of POM export increases with residence time, while that of DOM export declines. Thus, nutrient fertilization that favors larger, sinking phytoplankton (POM) enhances long-term carbon storage per nutrient drawn down, whereas shifts toward smaller, advected phytoplankton (DOM) reduce the long-term carbon sequestration efficiency. Thus, the success of marine CDR strategies depends not only on surface productivity but also on the balance between POM and DOM production and their respective remineralization rates.

### Conclusions.

Our results highlight that the global residence-time distributions of carbon and phosphorus need not coincide—a nuance often overlooked in discussions of marine biogeochemical cycles. The model sensitivity to poorly constrained fast cycling fluxes also demonstrates a critical need for empirical studies aimed at better quantifying the remineralization rates and bioavailability of organic matter. While much of the current research focuses on biological carbon fluxes, we emphasize the need to give greater attention to the cycling of other essential elements, especially when predicting how the long-term sequestration of carbon and nutrients in the ocean interior will respond to perturbations. This decoupling has important consequences for the efficacy of CDR strategies, such as ocean iron fertilization, which rely on enhancing surface productivity to sequester carbon in the deep ocean. Although such strategies may temporarily increase carbon export, our findings indicate that the long-term sequestration potential could be limited by the slow return of nutrients to surface waters, which could suppress future productivity and reduce the net removal of atmospheric CO_2_. Accounting for the timescales of both carbon and nutrient cycling will be critical for developing effective strategies to mitigate climate change through ocean-based carbon sequestration.

## Materials and Methods

### Biogeochemical Model.

We model the cycling of phosphorus, carbon, and oxygen in the ocean with an optimized biogeochemical inverse model based on that of Wang et al. (38). The model uses a data-constrained transport matrix from the ocean circulation inverse model (OCIM2; 39, 40). The model has a horizontal resolution of 2° × 2° and 24 vertical layers ranging in thickness from 36 m at the surface to 5,433 m at the deepest layer. The phosphorus cycling model solves for the equilibrium state of four reservoirs: dissolved inorganic phosphorus (DIP), particulate organic phosphorus (POP), dissolved organic phosphorus (DOP), and labile DOP (DOP_*l*_). The carbon cycle model solves for the equilibrium state of seven tracers: dissolved inorganic carbon (DIC), total alkalinity (ALK), particulate inorganic carbon (PIC), particulate organic carbon (POC), and three distinct pools of dissolved organic carbon (DOC) with varying lability. The semilabile pool has an optimizable temperature-dependent remineralization timescale, $1/\kappa_{C}$. The refractory pool (DOC_*r*_) has a much longer remineralization timescale. Production of labile DOC (DOC_*l*_), which has a rapid respiration timescale of 12 h, is adjusted to allow the model to match different satellite-based NPP products. In the main results presented here, the labile DOC pool was defined to elevate the model total organic carbon production to match the climatological NPP distribution computed from the carbon-based productivity model (CbPM; 24). We constrain the sum of these three DOC pools to best match a dataset of bulk DOC concentration measured in situ (41). Tracing three distinct DOC pools allows us to avoid making assumptions about the contribution of refractory DOC to the observed bulk DOC concentration.

We model organic matter production following the approach of Wang et al. (38), in which biological production of organic phosphorus is proportional to the modeled DIP concentration with an empirically derived uptake rate coefficient, γ, which varies with satellite-derived NPP and observed surface DIP concentrations. A spatially variable C:P ratio is used to convert organic phosphorus production to organic carbon production. The C:P stoichiometry of production varies spatially, according to the model DIP concentration as, $r_{C:P}={\left(cc\left[\mathrm{DIP}\right]+dd\right)}^{-1}$, where cc and dd are optimizable parameters (42).

We model the vertical attenuation of particulate organic carbon (POC) and phosphorus (POP) fluxes with the power law, $f\left(z\right)\propto z^{-b},$ following Martin et al. (26), where b is the particle attenuation coefficient, denoted $b_{C}$ and $b_{P}$ for carbon and phosphorus, respectively. Both coefficients are temperature-dependent and parameterized as,

$$b=b_{0}+b_{\theta }\cdot \bar{\theta },$$

where $\bar{\theta }$ is the normalized temperature, vertically averaged over the top three model layers (114 m). $b_{C_{0}}$, $b_{C_{\theta }}$, and $b_{P_{0}}$, $b_{P_{\theta }}$ are optimizable parameters controlling the flux attenuation profile of POC and POP, respectively.

The remineralization rate constants for semilabile DOC ($k_{C}$) and DOP ($k_{P}$) also vary spatially, with a $Q_{10}$ type temperature dependence,

$$\begin{array}{c} k=k_{d}\cdot Q_{10}^{\left(\frac{T-T_{0}}{10}\right)}, \end{array}$$

where T is the climatological water temperature from World Ocean Atlas 2018 (43), $k_{\mathrm{dC}}$ and $k_{\mathrm{dP}}$ are optimizable reference remineralization rate constants (s^−1^) at the reference temperature $T_{0}$ = 30 °C, and $Q_{10C}$ and $Q_{10P}$ are optimizable parameters controlling the strength of the temperature dependence.

### Parameter Optimization.

The model includes 21 adjustable parameters (*SI Appendix*, Table S1), which are constrained using global datasets of DIP, total alkalinity, DIC, O_2_, DOC, and DOP (27, 41, 44) through a Bayesian inversion procedure, as described by Wang et al. (38). Our objective function is slightly modified from Wang et al. (38) by the inclusion of an additional term to constrain the model DOP to observed concentrations.

We solve for the most probable parameter values by minimizing the negative logarithm of the posterior probability function, defined as

$$\begin{array}{cc} f=\frac{1}{2} & (e_{\mathrm{DIP}}^{\prime }W_{\mathrm{DIP}}e_{\mathrm{DIP}}+e_{\mathrm{DOP}}^{\prime }W_{\mathrm{DOP}}e_{\mathrm{DOP}}+e_{\mathrm{DIC}}^{\prime }W_{\mathrm{DIC}}e_{\mathrm{DIC}}\\ & +e_{\mathrm{DOC}}^{\prime }W_{\mathrm{DOC}}e_{\mathrm{DOC}}+e_{\mathrm{ALK}}^{\prime }W_{\mathrm{ALK}}e_{\mathrm{ALK}}+e_{O_{2}}^{\prime }W_{O_{2}}e_{O_{2}})\\ & +\;\text{const.}, \end{array}$$

where $e_{x}$ are vectors whose elements contain the difference between modeled and observed concentrations of tracer, x, and $W_{x}$ is a precision matrix, containing the volume of the model grid boxes.

### Sequestration-Time-Partitioned Distribution Functions.

To solve for the sequestration time partitioned regenerated carbon production, we time-step the model forward using a trapezoid time integration scheme. For these runs, the model is initialized with a spatially distributed pulse of regenerated carbon. The spatial pattern of this pulse is given by the organic matter remineralization rate in the steady-state model. This regenerated carbon is then transported using the steady-state transport matrix operator until it enters the model euphotic zone, where the regenerated carbon is removed via a fast restoring operator, which restores the surface concentration of regenerated carbon to zero with a restoring timescale of (1/500) years. For each time step, we calculate the globally integrated total amount of regenerated carbon and nutrients remaining in the ocean interior. The ratio of the globally integrated regenerated carbon to regenerated phosphorus is plotted as a function of residence time in Fig. 1*C*. We track the regenerated carbon and nutrients originating from each organic tracer separately using separate initial pulses for the remineralization of POC, POP, DOC, DOP, DOC_*l*_, DOP_*l*_, and DOC_*r*_. The residence time distribution of these tracers is shown in Fig. 1*A* for biogenic carbon and Fig. 1*B* for biogenic phosphorus. The total regenerated carbon is the sum of all regenerated carbon remineralized from POC, DOC, DOC_*l*_, and DOC_*r*_. Likewise, the total regenerated phosphorus is the sum of contributions originating from both particulate and dissolved organic phosphorus.

Note that here residence time is defined as the interval between remineralization and return of carbon or nutrients to the surface, thereby excluding the sinking interval from the euphotic zone to the depth of remineralization. As a result, our residence times underestimate the full surface-to-surface transit time of organic-matter production. This simplification has minimal impact, as the time from production to remineralization is short compared to the residence time for all organic tracers except recalcitrant DOC and DOP, which contribute very little to the total sequestration fluxes.

## Supplementary Material

Appendix 01 (PDF)

## Data Availability

Model output from the inverse model, supporting data to run the model, and model code are available at https://doi.org/10.6084/m9.figshare.29549363 (45).
