# Supplementary material for: Decoupled timescales of organic carbon and phosphorus recycling in the global ocean
Source: Proc Natl Acad Sci U S A. 2026 Feb 17;123(8):e2514991123. doi: 10.1073/pnas.2514991123 (PMC12933148; doi:10.1073/pnas.2514991123)
Supplement: Supplementary file 1 — Appendix 01 (PDF) [file pnas.2514991123.sapp.pdf]

# Supporting Information for

## Decoupled timescales of organic carbon and phosphorus recycling in the global ocean

**Megan R. Sullivan, François W. Primeau, Hojong Seo, Judith Camps-Castellà, Keisuke Inomura, and Adam C. Martiny**

**Megan R. Sullivan.**
**E-mail: meganrs@uci.edu**

### This PDF file includes:

Figs. S1 to S3
Table S1
SI References

**Table S1. Optimized Parameter Values with Descriptions and Units**

| Parameter | Value | Description | Units |
|---|---|---|---|
| $\sigma_P$ | $2.66 \times 10^{-2}$ | Fraction of organic production routed to semi-labile DOP | - |
| $k_{\mathrm{dP}}$ | $7.86 \times 10^{-9}$ | Semi-labile DOP remineralization rate constant at 30°C | $\mathrm{s}^{-1}$ |
| $Q_{10P}$ | $5.73$ | Temperature dependence of semi-labile DOP remineralization rate | - |
| $b_{P_0}$ | $4.59 \times 10^{-1}$ | Exponent of POP flux attenuation profile at 0°C | - |
| $b_{P\theta}$ | $1.07$ | Temperature dependence of POP flux attenuation profile exponent | $°\mathrm{C}^{-1}$ |
| $\alpha$ | $1.07 \times 10^{-8}$ | Coefficient for NPP scaling | $\mathrm{s}^{-1}$ |
| $\beta$ | $8.60 \times 10^{-1}$ | Exponent for NPP scaling | - |
| $\sigma_C$ | $6.93 \times 10^{-2}$ | Fraction of organic carbon production routed to semi-labile DOC | - |
| $k_{ru}$ | $4.75 \times 10^{-16}$ | Remineralization rate constant for $DOC_r$ in euphotic zone | $\mathrm{s}^{-1}$ |
| $k_{rd}$ | $2.73 \times 10^{-12}$ | Remineralization rate constant for $DOC_r$ in the dark ocean | $\mathrm{s}^{-1}$ |
| $1-\eta$ | $2.90 \times 10^{-2}$ | Fraction of DOC that is transferred to the refractory pool | - |
| $b_{C_0}$ | $3.60 \times 10^{-1}$ | Exponent of POC flux attenuation profile at 0°C | - |
| $b_{C\theta}$ | $1.45$ | Temperature dependence of POC flux attenuation profile exponent | $°\mathrm{C}^{-1}$ |
| $d$ | $5.40 \times 10^{3}$ | e-folding length scale for PIC flux attenuation | $\mathrm{m}$ |
| $k_{dC}$ | $5.43 \times 10^{-9}$ | Semi-labile DOC remineralization rate constant at 30°C | $\mathrm{s}^{-1}$ |
| $Q_{10C}$ | $1.46$ | Temperature dependence of semi-labile DOC remineralization | - |
| $r_{\mathrm{RR},0}$ | $1.90 \times 10^{-2}$ | Rain ratio ($CaCO_3$ to POC production ratio) | - |
| $r_{\mathrm{Si}}$ | $1.57 \times 10^{-1}$ | Si dependence of rain ratio | - |
| $\mathrm{cc}$ | $1.36 \times 10^{-3}$ | Coefficient of [DIP] in C:P uptake equation | $\frac{\mathrm{molP}}{\mathrm{molC}\mu\mathrm{M}^{-1}}$ |
| $\mathrm{dd}$ | $7.46 \times 10^{-3}$ | Constant coefficient in C:P uptake equation | $\frac{\mathrm{molP}}{\mathrm{molC}}$ |
| $\mathrm{r}_{\Sigma-\mathrm{O_2}:\mathrm{C}}$ | $1.82$ | Respiration quotient, molar ratio of $O_2$ utilized to C respired | - |

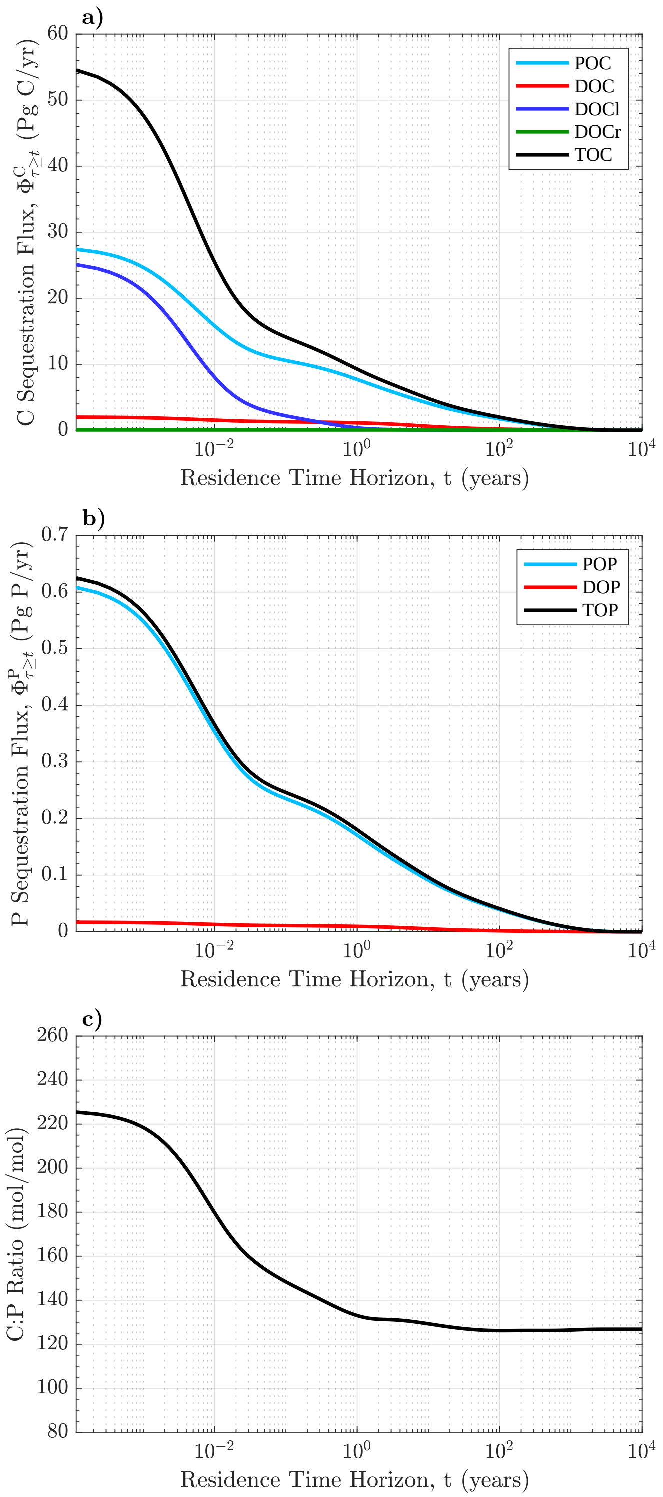


**Fig. S1. Sequestration fluxes in a sensitivity test with equal carbon and phosphorus remineralization rates.** For this sensitivity test, the parameters controlling the flux attenuation of particulate organic matter and the remineralization rate of semilabile dissolved organic matter are set equal to spatially constant values: $b_C = b_P = 1.28$ and $k_C = k_P = 0.057$ y$^{-1}$. **a–b)** The biogenic sequestration fluxes, $\Phi^{C}_{\tau\geq t}$ and $\Phi^{P}_{\tau\geq t}$, defined as the global production of organic carbon (a) and phosphorus (b) whose regenerated inorganic forms remain in the interior for at least a threshold residence time, $t$ years. $\tau$ denotes the first-passage time of regenerated inorganic carbon ($DIC_{bio}$) and phosphorus ($DIP_{bio}$)—that is, the time until their next surface contact—following Primeau (1). **c)** The ratio of the total carbon sequestration flux to the total phosphorus sequestration flux as a function of the residence time horizon. In (**a-b**), total organic carbon (TOC) and total organic phosphorus (TOP) sequestration fluxes (black) are the sum of remineralized particulate organic matter (light blue), semi-labile dissolved organic matter (red), labile organic matter (dark blue), and recalcitrant dissolved organic matter (green) production. For a residence time horizon of $t = 0$, the TOC sequestration flux is equivalent to global net primary production (NPP), which is prescribed to match satellite-based climatological estimates from the CbPM algorithm (2).

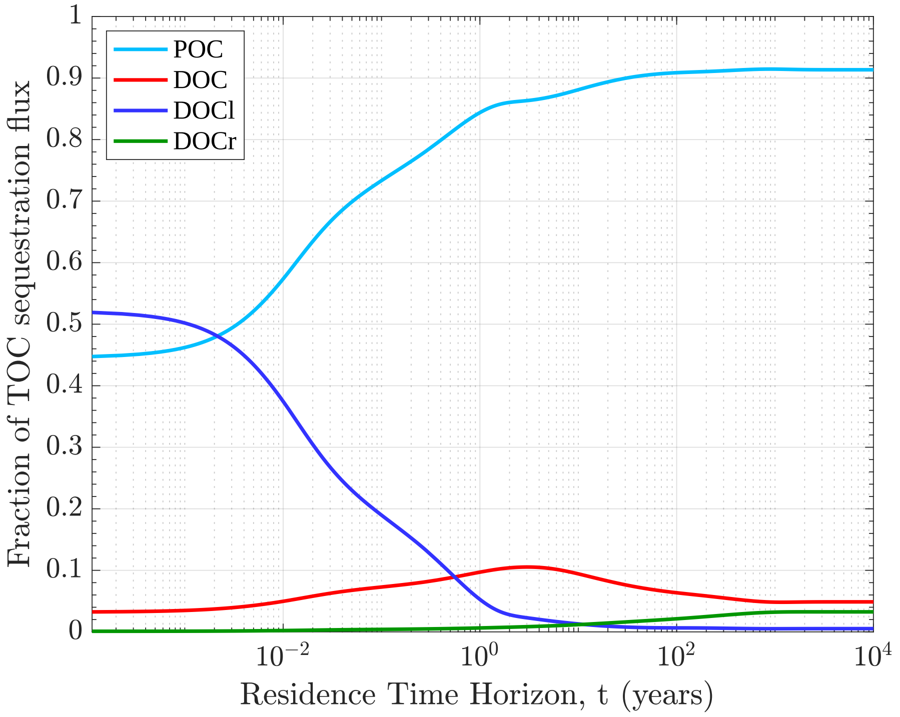


**Fig. S2. Fractional contributions of organic carbon pools to the total organic carbon sequestration flux,** $\Phi^{\mathrm{TOC}}_{\tau\geq t}$**.** The sequestration flux is defined as the global production of organic carbon whose regenerated inorganic form remains in the ocean interior for at least a threshold residence time $t$ (years); $\tau$ denotes the first-passage time of regenerated inorganic carbon to the surface. At $t = 0$, $\Phi^{\mathrm{TOC}}_{\tau\geq 0}$ is equivalent to total organic carbon (TOC) production and is composed of 52% labile dissolved organic carbon ($\mathrm{DOC}_l$), 45% particulate organic carbon (POC), and 3% semi-labile dissolved organic carbon (DOC), with negligible production of recalcitrant DOC ($\mathrm{DOC_r}$). By a residence time horizon of 1 year, $\mathrm{DOC}_l$ supplies only 5% of the sequestration flux, while POC contributes 85%. At century timescales, more than 90% of the sequestration flux, $\Phi^{\mathrm{TOC}}_{\tau\geq 100\,\mathrm{y}}$, originates from POC.

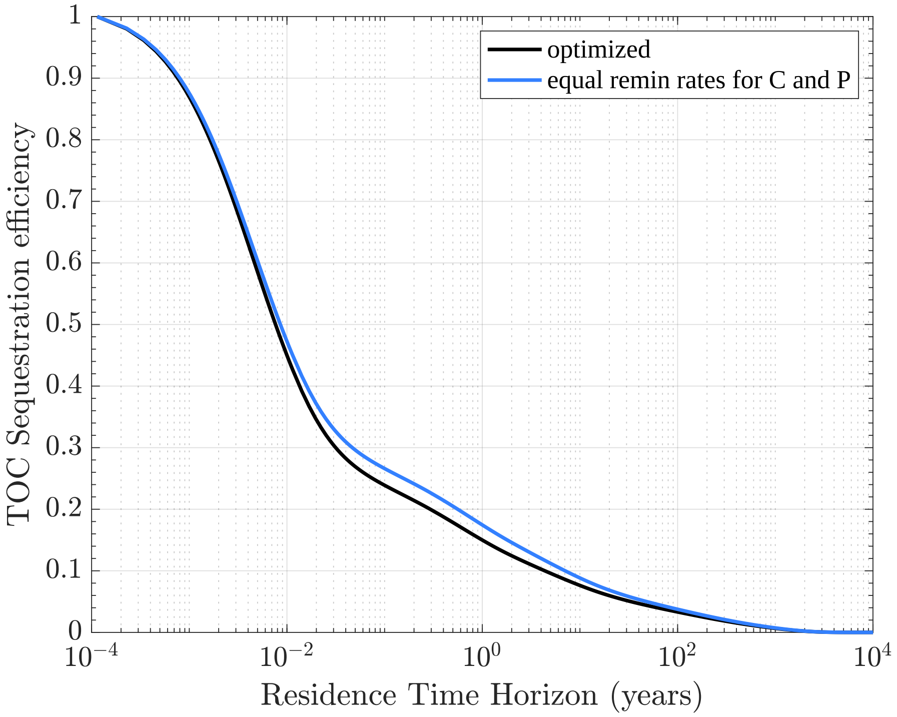


**Fig. S3. Time-dependent TOC sequestration efficiency in a sensitivity test with equal carbon and phosphorus remineralization rates.** The total organic carbon (TOC) sequestration efficiency is defined as the fraction of TOC production that remains sequestered in the ocean interior for at least a given residence time horizon. Results are shown for the optimized model (black) and a sensitivity test in which organic carbon and phosphorus share identical remineralization parameters (blue). In the equal-remineralization model, the parameters controlling particulate organic phosphorus (POP) flux attenuation ($b_{P_0}$ and $b_{P\theta}$) and semi-labile dissolved organic phosphorus (DOP) remineralization ($k_{dP}$ and $Q_{10P}$) are set equal to those of organic carbon at every location. The higher sequestration efficiency in the equal-remineralization case reflects enhanced phosphorus recycling, which supports greater particulate organic matter production and deeper carbon export. Differences are most pronounced at intermediate timescales: the optimized model, in which organic phosphorus remineralizes more slowly than organic carbon, exhibits 13.7%, 11.7%, 11.2%, and 9.9% lower TOC sequestration efficiency at 10, 100, 200, and 1000 years, respectively, compared to the equal-remineralization case.

## References


1. F Primeau, Characterizing transport between the surface mixed layer and the ocean interior with a forward and adjoint global ocean transport model. *J. Phys. Oceanogr.* **35**, 545–564 (2005).
2. T Westberry, MJ Behrenfeld, DA Siegel, E Boss, Carbon-based primary productivity modeling with vertically resolved photoacclimation. *Glob. Biogeochem. Cycles* **22**, 2024 (2008).
